# An Improved FAST Algorithm Based on Image Edges for Complex Environment

**DOI:** 10.3390/s22197127

**Published:** 2022-09-20

**Authors:** Cunzhe Lu, Xiaogang Qi, Kai Ding, Baoguo Yu

**Affiliations:** 1 School of Mathematics and Statistics, Xidian University, Xi’an 710071, China; 2 Xi’an Key Laboratory of Network Modeling and Resource Scheduling, Xi’an 710071, China; 3 Science and Technology on Near-Surface Detection Laboratory, Wuxi 214000, China; 4 State Key Laboratory of Satellite Navigation System and Equipment Technology, Shijiazhuang 050051, China

**Keywords:** visual SLAM, point and line feature, FAST algorithm, image edge, adaptive Canny

## Abstract

In complex environments such as those with low textures or obvious brightness changes, point features extracted from a traditional FAST algorithm cannot perform well in pose estimation. Simultaneously, the number of point features extracted from FAST is too large, which increases the complexity of the build map. To solve these problems, we propose an L-FAST algorithm based on FAST, in order to reduce the number of extracted points and increase their quality. L-FAST pays more attention to the intersection of line elements in the image, which can be extracted directly from the related edge image. Hence, we improved the Canny edge extraction algorithm, including denoising, gradient calculation and adaptive threshold. These improvements aimed to enhance the sharpness of image edges and effectively extract the edges of strong light or dark areas in the images as brightness changed. Experiments on digital standard images showed that our improved Canny algorithm was smoother and more continuous for the edges extracted from images with brightness changes. Experiments on KITTI datasets showed that L-FAST extracted fewer point features and increased the robustness of SLAM.

## 1. Introduction

Simultaneous localization and mapping (SLAM) is a challenging problem in the field of mobile robots, which has been widely studied for more than 20 years [1]. People use different technologies to improve the autonomous navigation and self-exploration abilities of robots, requiring them to navigate autonomously in the environment without any interference [2]. Successful navigation requires the robot to have a good understanding of its environment, and an ability track its position in the environment stably and accurately [3]. The localization of a robot is usually described as the state of it, including position and posture in the relevant map [4]. Some technologies used to solve this problem, such as ultra-wide band (UWB) [5,6], Bluetooth [7,8], etc., need to rely on the previous resource deployment for environmental scenes. However, a fully intelligent mobile robot is often required to work normally in an unknown environment. In recent years, visual SLAM (vSLAM) with a camera as the only sensor has become a hot research topic in the robot field due to its low cost and high practicability [9,10,11].

A vSLAM system is based on the sequence frames obtained by visual sensors, and uses image information and models to estimate a robot’s pose. In the past few decades, a relatively mature vSLAM framework has been developed, which can be divided into four parts: information collection, front end, back end and mapping [9].

The front end of a vSLAM system is more related to the field of computer vision, which mainly calculates the relative pose between adjacent frames. According to whether it extracts image features or not, vSLAM front ends can be divided into two kinds, direct method and feature point method. The direct method, using algorithms such as parallel tracking and mapping [3], large-scale direct monocular SLAM [12], semi-direct visual odometry [13], direct sparse odometry [14], etc., does not need to calculate the key points and descriptors, which estimate the camera motion and the projection of points according to the pixel gray information to minimize the photometric error. This is regarded as a loss minimization problem, and the pose estimation is iteratively optimized through the loss function. The direct method can work well in scenes with brightness changes. However, it strongly depends on the assumption that the gray scale is invariant between adjacent frames, which is difficult to establish in reality. The feature point method usually adopts scale-invariant feature transform (SIFT) [15], speeded-up robust features (SURF) [16], features from accelerated segment test (FAST) [17] and other algorithms to extract image point features. Then, the random sample consensus (RANSAC) [18] algorithm is usually used to match point features and eliminate wrong matching. Finally, an extended Kalman filter (EKF) [19], bundle adjustment (BA) [20] and other methods are used to estimate the relative pose by minimizing the reprojection error. The most common system is oriented FAST and rotated BRIEF SLAM (ORB-SLAM) [21,22].

ORB-SLAM uses FAST to extract point features. The extraction speed is very fast: it can extract a large number of point features. However, its disadvantages are also obvious. For some lightweight devices, too many point features will lead to longer mapping time. In addition, in some common complex environments, such as scenes with low texture or brightness changes, it is difficult to extract enough representative point features, and the extracted point features are not enough to estimate the location information, which greatly limits the performance of ORB-SLAM. Even in the above environment, line features still have good performance, because the line feature extraction method led by line segment detector (LSD) [23] is mainly based on the gradient of each pixel. However, in most of the current research, line feature extraction and matching consume a lot of time. Most work only to extract features in the key frame as landmarks and to use in back-end optimization. In addition, for images with strong light and shadow areas at the same time, it is difficult to extract the features of the shadowed part.

An important step in extracting line features is the calculation of image edges. Image edge extraction is mainly the measurement, extraction and location of gray changes. The main idea of edge extraction is to use the edge enhancement operator to highlight the local edge in the image, then define the “edge intensity” of the pixel, and extract the edge point set by setting the threshold. Common edge extraction algorithms include differential operator, Laplace operator [24], Canny operator [25], etc. Canny operator is not easily disturbed by noise and can extract weak edges, but it also has the shortcomings of the above line feature extraction: it cannot perform good edge extraction to adapt to brightness changes. In addition, the Canny algorithm denoises the original image by Gaussian filtering before operation, which leads to the extraction of many non-existent weak edges.

In order to solve the above problems, we aimed to improve ORB-SLAM. The design idea was to add the consideration of line features to the strategy of point feature extraction, and use a line-intersection FAST (L-FAST) algorithm based on image edges, which is more sensitive to the intersection of line segments in the image. The main contributions are as follows.

- Improve traditional Canny algorithm. Our algorithm uses the improved median filter [26] to replace the Gaussian filter for denoising, so as to avoid the blurring of the edge. It uses the gradient extraction method in the LSD algorithm to replace the Sobel algorithm, so as to suppress the influence of the gradient on the edge, and the proposed double adaptive dual-threshold to determine the edge.
- Improve traditional FAST algorithm. Our algorithm is based on the edge image of the original image for feature extraction. The proposed method can effectively extract the intersection of two-line segments in a complex environment.

The rest of this paper is structured as follows: Section 2 reviews the related work used in this paper and the overview of our system. Section 3 describes in detail the improved Canny algorithm and the proposed L-FAST method. Section 4 presents experimental results using common datasets, followed by the conclusion and intended future work in Section 5.

## 2. Related Work and System Overview

This paper focuses on improving the image point features extraction method, which mainly used the edge image to extract the intersection of two lines in the image. L-FAST is based on the traditional Canny algorithm and ORB-SLAM. Therefore, this section first gives a brief introduction to them, then an overview of L-FAST.

### 2.1. Introduction of Traditional Canny Algorithm

**Figure 1:**
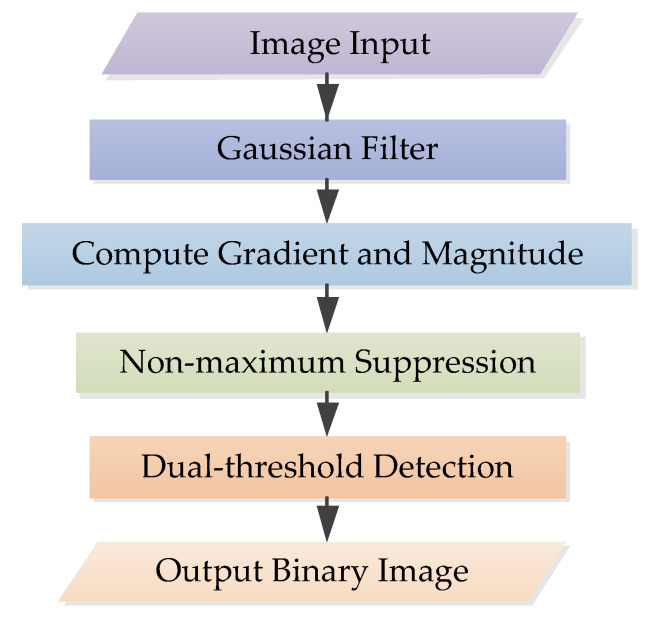
Flowchart of the traditional Canny algorithm.

Traditional Canny algorithm is a multistage algorithm, as shown in the flowchart in Figure 1. The Canny operator is applied to define image edge features as much as possible while reducing image data size. First, the image is denoised by Gaussian filter. Then, gradients in the horizontal, vertical and diagonal directions of each pixel are calculated. After that, each pixel in the gradient image is subjected to non-maximum suppression to determine whether the pixel point is preserved or suppressed. Finally, dual thresholds are used to connect the edges; the pixels below the low threshold are suppressed; above the high threshold they are judged as strong edge pixels, and between dual thresholds they are judged as weak edge pixels. For weak edge pixels, those far from the strong edge are suppressed, so as to obtain a binary image.

The effect of the traditional Canny algorithm relies on a manually set double threshold, and many scholars have optimized this problem. L. Wang et al. proposed an adaptive double-threshold-improved Canny algorithm based on added gradients [27]; Gu et al. adopted the improved Ostu algorithm [28] to adaptively generate Canny operator double thresholds according to the iterative threshold segmentation method [29]; Othman et al. used the Canny operator for static gesture segmentation using the proposed adaptive thresholding method [30]. However, since the traditional Canny operator does not consider the influence of illumination changes, these algorithms perform adaptive processing on the threshold, but they still do not perform well in scenes with large illumination changes. At present, common lighting processing methods include histogram equalization [31], gamma correction [32], low-light image enhancement [33], and multiscale Retinex model [34]. These methods have achieved good results, but the methods of extracting the enhanced image edge features are too time-consuming and do not provide the immediacy required by SLAM.

### 2.2. Introduction of Traditional ORB-SLAM and FAST Algorithm

ORB-SLAM calculates the main direction of point features on the basis of FAST, which adds rotation invariance to the BRIEF descriptors.

The main idea of FAST is that if a pixel differs greatly from enough pixels in its field, the pixel may be a corner [17]. However, due to the characteristics of FAST, point features are easy to gather together, so it is necessary to suppress the non-maximum value of point features to obtain the largest response value in the adjacent range. After extracting FAST point features, ORB-SLAM adds an image pyramid for scale invariance, and solves the problem that FAST does not have rotation invariance through the gray centroid method. After the extraction of oriented FAST point features, the BRIEF binary descriptor completes the calculation of the related points descriptor. So far, the whole process of ORB-SLAM has been completed.

Since ORB uses a fixed threshold, it is not effective in different scenarios and in the environment of sudden changes in brightness, and there are problems such as a small number of corner extractions, a low matching accuracy rate and poor adaptability. In response to this problem, many researchers have proposed different improvement schemes. M. Ying et al. proposed an improved thresholding algorithm based on gray clustering, which defines multiple adaptive thresholds to adapt to different lighting conditions [35]. Song et al. constructed a pyramid model with the help of a nonlinear filter, used the fast display diffusion algorithm to obtain a numerical solution, and solved the image coordinates, which gave good robustness in image scale, brightness and rotation [36]. Y. Xue et al. combined histogram equalization and dark channel prior theory to construct a color space with constant illumination, which ensured the amount of feature extraction, matching accuracy and efficiency [37]. However, due to the characteristics of the FAST algorithm, the corners it extracts have the characteristics of large quantity but low quality.

### 2.3. System Overview

**Figure 2:**
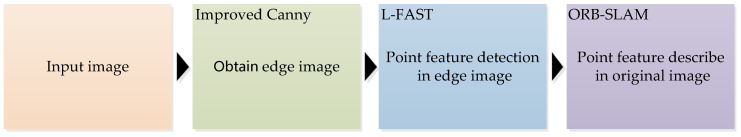
The flowchart of L-FAST.

The L-FAST algorithm proposed in this paper is mainly based on the Canny algorithm and ORB-SLAM. The flow of L-FAST is shown in the Figure 2, which is mainly composed of three parts: image edge extraction module, corner extraction module and point description module based on ORB-SLAM.

The Canny algorithm was mainly used and improved the image edge extraction module, which ensured that the edges in the image with brightness intensity changes could be extracted well. The point feature extraction module was mainly improved based on FAST to make it more sensitive to the intersection of two lines, which reduced the number of corner extractions. Finally, we used ORB-SLAM to describe the point features extracted by the above method.

For the image edge extraction module, we used the improved median filter to replace the Gaussian filter for denoising, so as to avoid the blurring of the edge. The gradient extraction method in the LSD algorithm was used to replace the Sobel algorithm to suppress the influence of the gradient on the edge. Finally, we carried out NMS, and used the proposed double adaptive dual threshold to determine the edge, so as to output the corresponding binary image.

**Figure 3:**
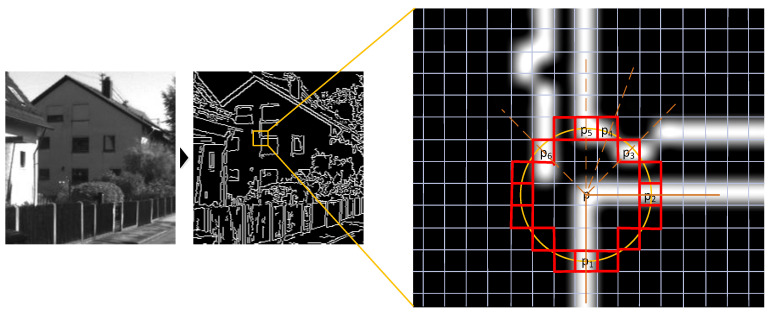
An example of the L-FAST idea.

For L-FAST, we noted that in the binary image, as shown in Figure 3 (the original image was obtained from the KITTI dataset [38]), if a point P is the intersection of two lines, and a circle is drawn with the center P, it will intersect with these two lines at least at two points, marked as $P_{1}$ and, then vector $PP_{1}$ and $PP_{2}$ on the line, respectively. According to this characteristic, we have improved the FAST algorithm. All the improvement processes are described in detail in Section 3.2 of this paper.

## 3. Improved FAST Processing

In this section, we first describe the improved Canny edge extraction algorithm, including image denoising, gradient calculation and dual-threshold judgment, then introduce L-FAST as proposed. After extracting point features from the edge image, the points are described on the original image. Since the description method used in this paper is consistent with that in ORB, it will not be described here.

### 3.1. Improved Adaptive Canny Method

**Figure 4:**
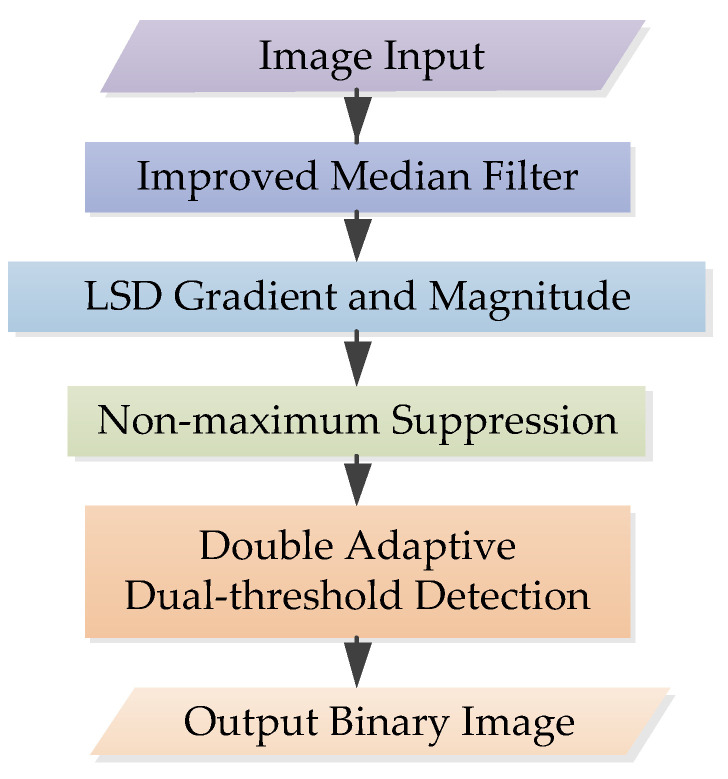
The flowchart of improved Canny algorithm.

The flowchart of the improved Canny algorithm used in this paper is shown in Figure 4. There are three main improvements: image preprocessing, gradient calculation and double adaptive dual-threshold calculation. Other parts are consistent with the traditional Canny algorithm, which will not be introduced. For details, see [25]. This section will show the improved methods in turn.

#### 3.1.1. Image Preprocessing

Before image edge extraction, in order to reduce the impact of noise on image edge extraction as much as possible, it is necessary to denoise the image. The traditional Gaussian filter smoothes the image. Although it can effectively reduce the impact of noise, the image smoothing has a certain negative impact on edge extraction, which leads to the original strong edge being weakened into a weak edge, or the original position that is not an edge being extracted as an edge. Since the L-FAST algorithm proposed is based on the edge image, it requires that the extracted image edge can be located at the original position of the image, and due to the noise in the image used for visual SLAM being usually short noise, median filtering can effectively remove such noise points. Therefore, we used the improved median filter to replace the Gaussian filter in the image preprocessing operation, which can effectively denoise and retain the image edge details.

The traditional median filter operates on each element in the image, but in fact, not each element contains a lot of noise. What we know is that for edge pixels, the pixel value in one direction in its neighborhood is close to its value, while for noise pixels, it is an outlier. Therefore, we can quickly judge the noise points according to this characteristic.

Suppose a point P(x,y) in the image has the value G(x,y). The pixel values of the upper, lower, left and right points of P are abbreviated as. $G_{n}\left(x,y\right),n=1,2,3,4$. Then, according to Equation (1), P can be distinguished into edges and noises.

(1) $$\{\begin{array}{l} \left|G\left(x,y\right)-G_{n}\left(x,y\right)\right|>\delta \cdot G\left(x,y\right),\;n=1,2,3,4,\;\;\;\;\;noisy\;point\\ else,\;\;\;\;\;edge\;point \end{array}$$

where δ is used to judge the similarity between P and adjacent pixels. If δ is too large, P will be missed as an edge, and if δ is too small, P will be misjudged as a noise. Here, take δ=0.1. After distinguishing the noisy pixels, carry out a 3×3 median filter on the noisy pixels. In addition, in the step of judging noise points, we can speed up the operation through the image integral graph.

#### 3.1.2. Calculate Gradient and Magnitude

In order to minimize the use of other pixels and reduce the dependence on gradients, this paper uses LSD gradients, using the four pixels below the right of each point to calculate, in order to record the brightness changes, so as to find the positions that may be edges. The calculation method of the gradient is shown in Equation (2).

(2) $$\begin{array}{c} g_{x}(x,y)=\frac{i(x+1,y)+i(x+1,y+1)-i(x,y)-i(x,y+1)}{2}\\ g_{y}(x,y)=\frac{i(x,y+1)+i(x+1,y+1)-i(x,y)-i(x+1,y)}{2} \end{array}$$

The gradient angle is computed as

(3) $$\arctan\left(\frac{g_{x}\left(x,y\right)}{g_{y}\left(x,y\right)}\right)$$

and the gradient maguitude as

(4) $$G\left(x,y\right)=\sqrt{g_{x}^{2}\left(x,y\right)+g_{y}^{2}\left(x,y\right)}.$$

#### 3.1.3. Double Adaptive Dual Threshold

After calculating the image gradient, the edge extracted only based on the gradient is still very fuzzy, because there are multiple gradient responses to the edge, so NMS is carried out in the gradient direction of each pixel. After NMS, the image will still cause some spurious responses due to noise and color changes. In order to solve these response issues, double threshold extraction is used to judge the strong and weak edges. The pseudo code of double threshold extraction is as follows Algorithm 1.
**Algorithm 1** Traditional double threshold extraction$1:\;if\;p_{i}\geq T_{1}:$$\;\;\;p_{i}\;is\;a\;strong\;edge$$2:\;else\;if\;p_{i}\geq T_{2}:$$\;\;\;p_{i}\;is\;a\;weak\;edge$3: else:$\;\;\;p_{i}\;should\;be\;suppressed$

Where $T_{1}$ and $T_{2}$ are high and low thresholds, respectively. In the traditional Canny algorithm, the high and low thresholds are set by personal experience. In practical engineering applications, due to various conditions, a manually set threshold will reduce the accuracy of edge extraction results. In addition, the traditional setting method has the problem that the edge is difficult to extract in the shadow when the whole image has obvious changes of brightness.

In order to solve the above problems, this paper subdivides the threshold into global and local thresholds. In terms of global threshold, we calculated the high threshold $T_{1}$ based on the Ostu method [28], with the low threshold $T_{2}$ set as half of $T_{1}$. For a local threshold, the local image is obtained through a sliding window, and the local high threshold $TL_{1}$ and local low threshold $TL_{2}$ are calculated through our proposed method. The pseudo code of the improved double adaptive dual-threshold extraction is as follows Algorithm 2.
**Algorithm 2** Improved double adaptive dual-threshold extraction$1:\;set\;t_{1}=\max\left(T_{1},TL_{1}\right),\;t_{2}=\min\left(T_{1},TL_{1}\right),\;t_{3}=\max\left(T_{2},TL_{2}\right),\;t_{4}=\min\left(T_{2},TL_{2}\right)$$2:\;if\;p_{i}\geq t_{1}:$$\;\;\;p_{i}\;is\;a\;strong\;edge$$3:\;else\;if\;t_{1}\geq p_{i}\geq t_{2}\;:$$\;\;\;p_{i}\;is\;a\;weak-strong\;edge$$4:\;else\;if\;t_{2}\geq p_{i}\geq t_{3}:$$\;\;\;p_{i}\;is\;a\;weak\;edge$$5:\;else\;if\;t_{3}\geq p_{i}\geq t_{4}:$$\;\;\;p_{i}\;is\;a\;weak-weak\;edge$6: else:$\;\;\;p_{i}\;should\;be\;suppressed$

So far, pixels marked as strong edges have been identified as edges, and edges below the low threshold have been suppressed. For weak-strong edges, weak edges and weak-weak edges, they may be extracted from real edges, or they may be caused by noise or color changes. We notice that the edges caused by noise are isolated. In addition, for weak-strong edges, the probability is extracted from the real edge, but because the real edge is in the global shadow part, the gradient is small and the edges are judged as weak-strong edges. Therefore, for a weak-strong edge, if it is connected with another weak-strong edge, the weak-strong edge will be retained as a real edge. For a weak edge, if it is connected with a strong edge, the weak edge will be retained, otherwise, it will be suppressed. For weak-weak edges, the cost of misjudging into strong edges is huge. Therefore, if one weak-weak edge is connected with another weak-weak edge, the weak-weak edge is discarded; otherwise it is treated as a weak edge. To sum up, the pseudo code for suppressing isolated edge points is as follows Algorithm 3.
**Algorithm 3** Suppressing isolated edge points$1:\;if\;p_{i}\;is\;a\;weak-strong\;edge\;and\;p_{i}\;is\;connected\;to\;a\;weak-strong\;edge:$$\;\;\;p_{i}\;is\;a\;strong\;edge$   else:$\;\;\;p_{i}\;should\;be\;treat\;as\;weak\;edge$$2:\;if\;p_{i}\;is\;a\;weak-weak\;edge\;and\;p_{i}\;is\;connected\;to\;a\;weak-weak\;edge:$$\;\;\;p_{i}\;should\;be\;suppressed$   else:$\;\;\;p_{i}\;should\;be\;treat\;as\;weak\;edge$$3:\;if\;p_{i}\;is\;a\;weak\;edge\;and\;p_{i}\;is\;connected\;to\;a\;strong\;edge:$$\;\;\;p_{i}\;is\;a\;strong\;edge$   else:$\;\;\;p_{i}\;should\;be\;suppressed$

The following gives the methods for obtaining values for global double thresholds $T_{1}$ and $T_{2}$, the sliding window and local double thresholds $TL_{1}$ and $TL_{2}$.

1. Ostu method to determine global double threshold

The Ostu method gives an initial threshold to divide the non-real edge part of a gradient image into pseudo edge and non-edge parts; that is, background and foreground. When the iterative segmentation operation obtains the maximum interclass variance, the segmentation threshold is the optimal segmentation threshold, set as $T_{1}$, and $T_{2}$ set as half of $T_{1}$. The calculation method is as follows:

The total average gradient of the image is:

(5) $$\mu =\omega_{0}\times \mu_{0}+\omega_{1}\times \mu_{1}$$

Ostu is:

(6) $$L=\omega_{0}{\left(\mu_{0}-\mu \right)}^{2}+\omega_{1}{\left(\mu_{1}-\mu \right)}^{2}$$

where $\omega_{0}$ and $\omega_{1}$ are the proportion values of front background pixels; $\mu_{0}$ and $\mu_{1}$ are the average gradients of front background pixels.

2. Adaptive local double threshold

In order to make the local threshold more reasonable, a sliding window of α×β size is used. Considering the gradual change of the image, the step size is set to $\frac{\alpha }{2}$ or $\frac{\beta }{2}$, and the whole image is traversed. Considering that the smaller the window is, the greater the time loss is, after the dataset verification, this paper takes the length and width of the window as the fifth part of the original image.

Calculate the statistical data in the local image, including the maximum, minimum and median. We define the average value V of the pixel gradient of the local image as follows:

(7) $$V=\frac{\max\left(image\right)+median\left(image\right)+\min\left(image\right)}{3}$$

Finally, we calculate the local high and low threshold through parameters α:

(8) $$TL_{1}=\min\left(255,\left(1+\alpha \right)\cdot V\right)$$

(9) $$TL_{2}=\max\left(0,\left(1-\alpha \right)\cdot V\right)$$

where α is used to change the percentage of gradient intensity statistics to determine the threshold. Good results in KITTI dataset show that α=0.35 is the best.

**Figure 5:**
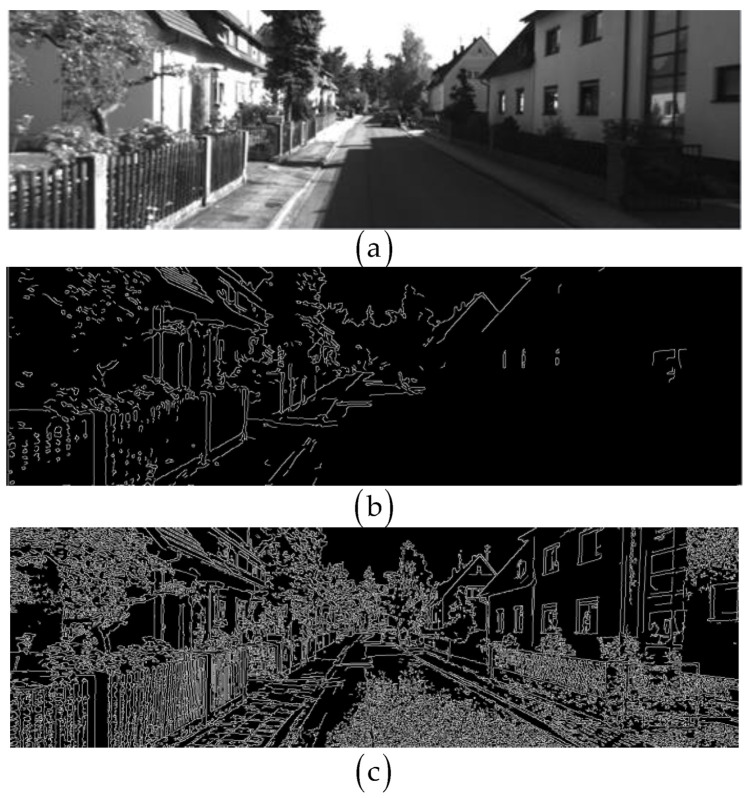
A demonstration of the effect of improving Canny, (**a**) is the original image, (**b**) is the edge image detected by Canny, (**c**) is the edge image detected by improved Canny.

The experience of applying our improved edge extraction algorithm is positive: it can be seen from Figure 5 [38] that the improved algorithm can extract better edges for images with varying brightness intensity, and can also improve the quality of image edge extraction.

### 3.2. Improved Point Features Extraction Method 

**Figure 6:**
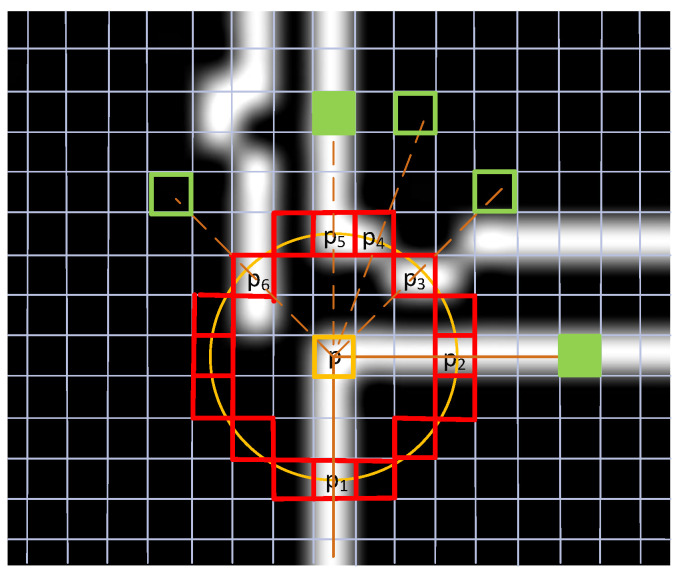
The principle of L-FAST algorithm to extract corners.

The edge image of the original image can be obtained from the previous steps, which is composed of some clearly demarcated lines in the image. Each point represents a point on the line. The main idea of L-FAST is that in an edge image, if the included angle of a point is not a flat angle, it is more likely to be a corner. Therefore, compared with other algorithms, the L-FAST algorithm is more sensitive to end points. Suppose that in the edge image, 255 represents the edge and 0 represents the background. Then, the steps of L-FAST algorithm are as follows, which can be seen from Figure 6.

Step 1 Note the current pixel is p, whose value is $I_{p}$; if $I_{p}$ is 255, then turn to Step 2; Otherwise, turn to Step 5.

Step 2 Draw a circle with p as its center and a radius of 3; the pixels passed by the circle are recorded as $p_{1},p_{2},p_{3},\cdots ,p_{16}$, turn to Step 3.

Step 3 Traverse the pixel values on the circle in turn. If there are two or more points with a value of 255 and p is not on the line of the only two points, note the pixels with value of 255 as $t_{1},t_{2},\cdots ,t_{n}$, turn to Step 4. Otherwise, turn to Step 5.

Step 4 Find the symmetric point $pt_{1},pt_{2},\cdots ,pt_{n}$ of p about $t_{1},t_{2},\cdots ,t_{n}$. If $pt_{i}$ is 0, check the value of eight points around $pt_{i}$, if there are more than three points equal to 255, mark $pt_{i}$ as 255. When there are two or more $pt_{i}$ values of 255, p is a corner. Otherwise, p is not a point feature, turn to Step 5.

Step 5 Turn to Step 1, until the last pixel of the image.

The frequent phenomenon of L-FAST corner clustering can be controlled by the NMS algorithm to reduce the clustering problem. Since the corners will also be lost when the image scale changes, the scale feature can also be added by building pyramids of images with different resolutions and extracting corners of multi-layer images. For scale, the original image is zoomed out and shrunk many times to obtain images with different resolutions. The matching algorithm is used to match the corners extracted by L-FAST in different layers of the image, and the sum of the point features extracted on each image layer of the pyramid is used as the point feature set of the image, so as to achieve scale invariance. So far, the L-FAST algorithm has been described.

## 4. Results and Discussion

The experiment was carried out on a personal computer equipped with AMD R5 5600 H CPU, 4.2 ghz × 6, and 16 GB RAM. The digital standard images Lena, cameraman, peppers and terraux [38] were used to verify the image data of the improved Canny algorithm. KITTI datasets 00–10 sequence images were used as image data to verify the performance of the L-FAST algorithm [39]. The experiment included two parts, one to evaluate the improved Canny algorithm, the other to evaluate L-FAST algorithm.

### 4.1. Evaluation of Improved Canny Method

In order to estimate the smoothing effect of the improved Canny edge extraction algorithm and the effectiveness of edge extraction, we added 5% ‘salt and pepper’ noise to the experimental image. In Experiment 1, the effectiveness of the improved median filter algorithm was compared with the Gaussian filter through subjective analysis and quantitative data analysis. In Experiment 2, the improved Canny edge extraction algorithm was quantitatively analyzed with the traditional Canny algorithm through the evaluation index in [40].

#### 4.1.1. Experiment 1

**Figure 7:**
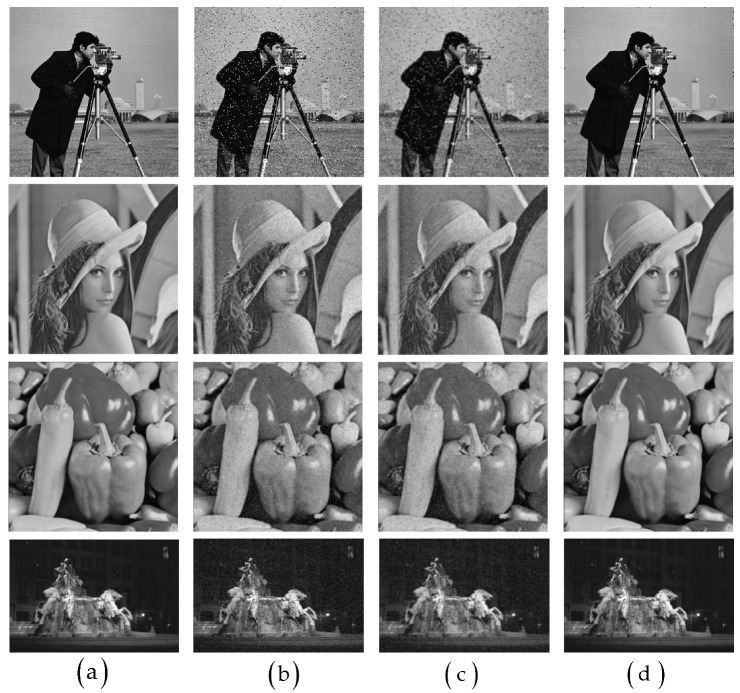
Comparison diagram of proposed Median Filter and Gaussian Filter, (**a**) is the original image, (**b**) is the image with 5% salt and pepper noise, (**c**,**d**) are the denoising results of Gaussian Filter and proposed Median Filter respectively.

In order to determine the effectiveness of the improved median filter, α=0.8 Gaussian filter was used for comparison, and the comparison results are shown in Figure 7 [38].

It can be found from the comparison results that the effect of Gaussian filter was far inferior to that of the improved median filter. The improved median filter effectively removed noise points and provided a good support for subsequent edge extraction. We used the peak signal-to noise ratio (PSNR) and signal-to-noise ratio (SNR) [41] to quantitatively evaluate the image. The larger the SNR and PSNR values were, the smaller the image distortion. The extraction results are shown in the Table 1. The quantitative analysis results also show the rationality of the proposed filter.

#### 4.1.2. Experiment 2

**Figure 8:**
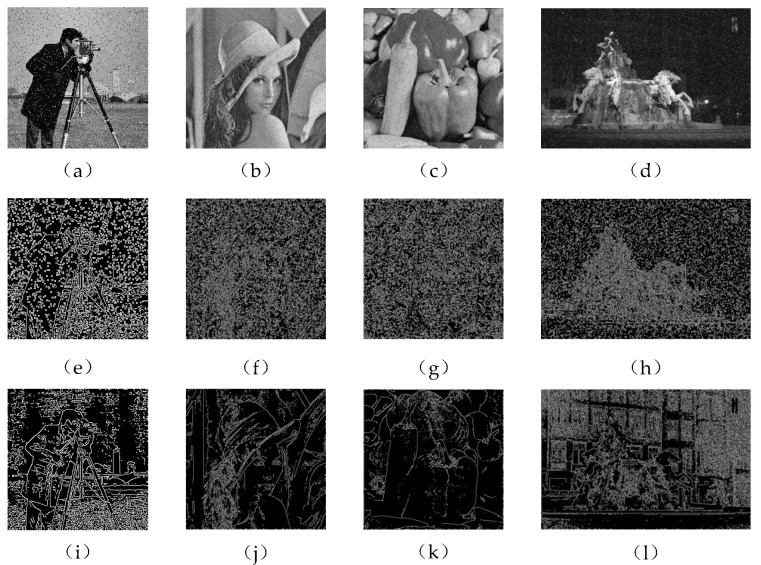
Comparison diagram of improved Canny algorithm and traditional Canny algorithm, (**a**–**d**) are the noisy images, (**e**–**h**) are the edge detection results using Canny respectively, (**i**–**l**) are the edge detection results using proposed method respectively.

In order to confirm the effectiveness of the improved Canny algorithm, the traditional Canny algorithm with α=0.8 and 200 and 100 (high and low) thresholds was used to compare the four images polluted by noise in Experiment 1. Figure 8 [39] shows different background experimental images of salt and pepper noise pollution.

Quantitative analysis was carried out according to the evaluation indicators in [40], and the total number of edge points (A), 4-neighborhood (B) and 8-neighborhood (C) values in the image were counted. The practicality and image edge continuity of this algorithm and Canny operator are expressed by ratio. The better the continuity of the image edge, the closer the ratio of C/B is to 0. C/A represents the smoothness of the image.

It can be seen from Table 2 that the edge extraction effect of the proposed algorithm in the case of 5% salt and pepper noise was much better than that of the traditional Canny algorithm, and it had certain anti-interference ability for the shadow part of the image.

### 4.2. Evaluation of L-FAST Method

In order to study the performance of L-FAST, this section first analyzes the number and time values of point feature extraction. Then, it is applied to ORB-SLAM to replace the FAST module in the ORB, and other modules remain unchanged. It is compared with the traditional ORB to analyze and compare the performance of L-FAST.

**Figure 9:**
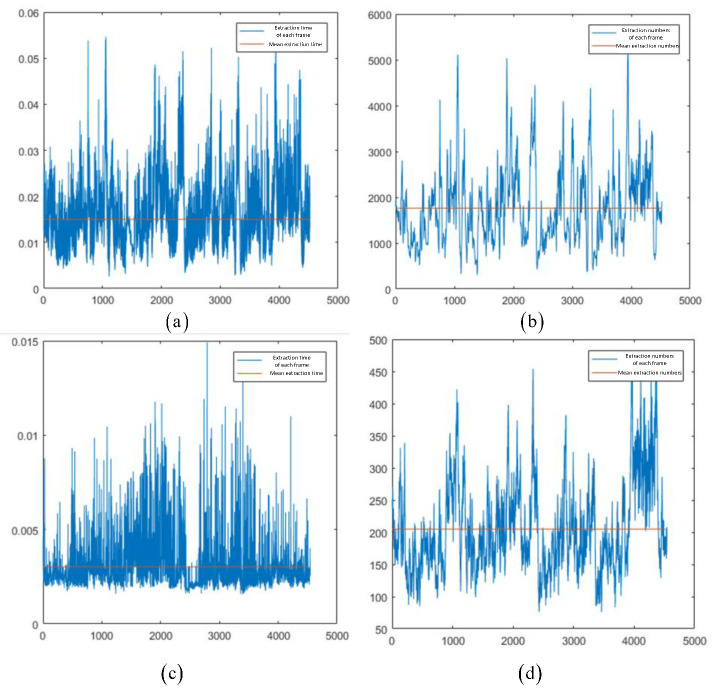
FAST and L-FAST extraction times and extracted number of point features diagrams for KITTI-00. The images (**a**,**b**) were taken by FAST; (**c**,**d**) were taken by L-FAST. The vertical axes of (**a**,**c**) represents time in seconds; the vertical axes of (**b**,**d**) represent numbers.

This section describes the use of the KITTI dataset 00–10 sequences diagrams to conduct the experiment. The accelerated ORB-SLAM was used for comparative experiments. Taking the KITTI dataset 00 sequence as an example, the image sequence was introduced into FAST and L-FAST algorithms, respectively, and the time consumed in extraction and the number of successfully extracted feature relationships was recorded. The visual data are shown in Figure 9.

We can see that the average extraction time of L-FAST was 3 ms, while the average extraction time of FAST was 15 ms. The average number of point features extracted by FAST and L-FAST was 1763 and 205, respectively. In terms of time, the extraction time of L-FAST was slightly better than that of FAST. While ensuring the success rate of frame matching, the number of point features was significantly reduced.

**Figure 10:**
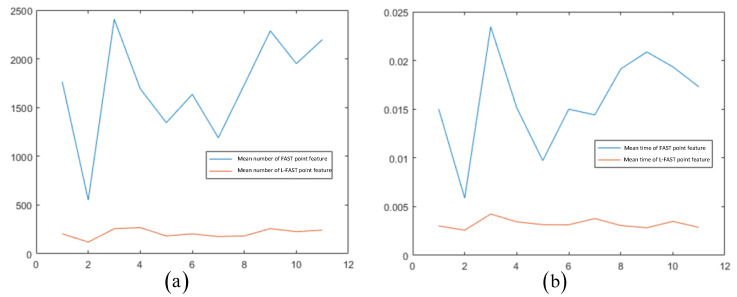
The average time (**a**) and number (**b**) results of each sequence.

In order to deeply compare the effects of different sequence sets, the same method was used to run 01–10 sequence datasets, respectively. The average time and number results of each sequence are shown in Figure 10.

The results of each sequence show that compared with FAST, L-FAST took less time to extract, and the number of extracts was relatively stable. In the case of less texture, L-FAST performed well. In order to deeply analyze the feasibility of specific applications in the system, the impact of FAST and L-FAST on the positioning performance of the simulation system was explored. The KITTI-05 dataset sequence was run to conduct the experiment. The images in the sequence were introduced into the simulation system using FAST and L-FAST, respectively, and their motion trajectories recorded. The absolute trajectory error was used to analyze the trajectory, and the root mean square error (RMSE) indexes of the two front ends were mainly compared. KITTI-05 sequence was composed of 2761 × 2 track photos, with a distance of about 3 km, tracking within 287.5 s, and the trajectory contained many loops.

**Figure 11:**
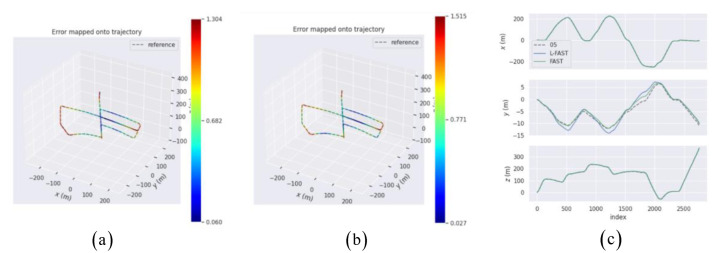
The motion trajectory ((**a**) is FAST and (**b**) is L-FAST) and error comparison (**c**) diagrams of two algorithms.

The motion trajectories are shown in Figure 11, including the trajectories of the simulation system using FAST and L-FAST, as well as the three-axis error comparison diagrams of different algorithms of sequence 05.

It can be seen from Figure 11 that the trajectory of L-FAST had smaller deviations in some areas than FAST. Further analysis of xyz three-axis error shows that the trajectory of L-FAST was basically consistent with the real trajectory of the KITTI-05 sequence on the xz axis, while the trajectory of L-FAST on the y axis had a slightly larger deviation than that of FAST.

In order to further analyze the performance of L-FAST, the quantitative analysis of each trajectory and the calculated absolute trajectory error (ATE) was carried out for each trajectory in the diagram, and the relevant indicators of error are given in Table 3.

**Figure 12:**
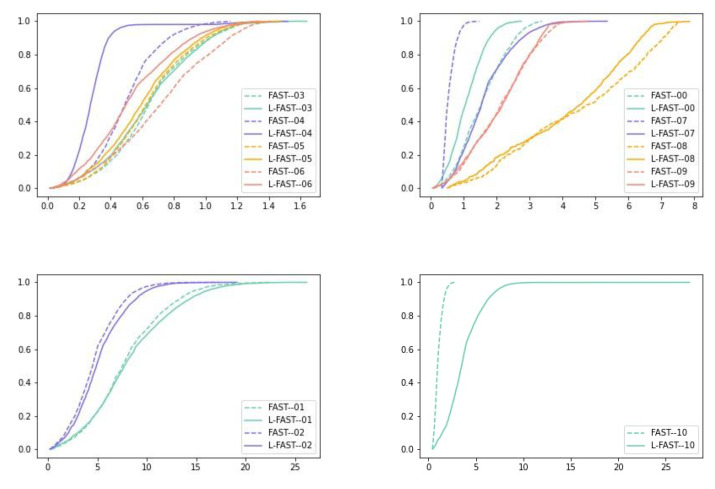
The CDFs of each sequence.

It can be seen from Table 3 that compared with FAST, the ATE of L-FAST decreased on the whole, but the improvement was not great. According to preliminary judgment, in the scene with many online features, L-FAST was better than FAST locally. Considering the conclusion drawn in Figure 11, L-FAST was obviously not good at the y axis calculations, which related to pitch angle, and the overall positioning accuracy of L-FAST on the xz axis was slightly better than that of FAST. In order to further analyze the reasons for the poor performance of the L-FAST algorithm on the y axis, the images of KITTI-00 to KITTI-10 sequences were run for comparison and analysis with FAST, and the time spent on each sequence was recorded to explore its time complexity. Table 4 shows the RMSE, min and max errors of ATE for sequence from 00 to 10, respectively. Table 5 shows the running time of each frame of the corresponding sequences, and the cumulative distribution functions (CDFs) of each sequence are shown respectively in Figure 12.

It can be seen from the error results of ATE and the CDFs that there was little difference between the two algorithms on 01, 02, 03, 05 and 09 sequences. The L-FAST algorithm performed better on 00, 04 and 08 sequences, but the performance of 07 and 10 sequences was far worse than that of FAST, which made a large error in some frames. By viewing the picture streams of each sequence in KITTI datasets, the car-following phenomenon occurred many times in the 07 sequence, and the white vehicle appeared for a long time in the 10 sequence. We judged that the quality of corners extracted by L-FAST was far less than that of FAST in these two sequences, due to it being easily detected as the intersection of the two lines from vehicles, which undoubtedly leads to a huge drift of some frames. In addition, for 01, 02, 05, 07, 09 and 10 sequences, it can be seen that the acquisition vehicles had a gentle slope process up and down. Among them, 01, 02, 07 and 10 sequences had a large slope change, while 05 and 09 sequences had a small slope change. The CDF images show that L-FAST was slightly less robust than FAST; this may be due to L-FAST using fewer corners. Therefore, we can conclude that L-FAST does not perform well on the y axis, and has certain robustness for small slopes. For 04 and 08 sequences images, the left-right motion of the camera caused by vehicle lane changes occurred many times during operation, but L-FAST still performed well. Therefore, we can conclude that L-FAST has strong robustness to parallel motion.

In terms of time, the matching time of each frame of L-FAST was about twice that of FAST. For 01, 03 and 06 sequences, it can be seen that their textures are relatively few, their brightness is uniform, and most of them are roads, so it took less time to extract edges. For 08 and 10 sequences, white vehicles exist in the image for a long time, resulting in uneven image brightness and increasing the time of edge extraction.

To sum up, L-FAST achieved similar performance to FAST in a general environment by using a small number of feature points. The proposed algorithm greatly improves the quality of feature points. The performance of L-FAST was poor in the environment with slope and abnormal car-following. L-FAST was robust in the left-right moving environments.

## 5. Conclusions

In this paper, we describe improvements to the traditional ORB-SLAM. We proposed a new point feature extraction criterion based on image edges. Compared with the traditional FAST algorithm, the L-FAST algorithm was more sensitive to the intersection of lines in the image. Simulation results show that the L-FAST algorithm significantly reduced the number of point features extracted while ensuring the same accuracy as the FAST algorithm, which is conducive to reducing the performance requirements of mapping in some lightweight devices. In addition, aiming at the problem that it is difficult to extract edges in the dark areas of an image with uneven brightness, we have improved the traditional Canny algorithm, which can effectively reduce the impact of uneven brightness.

However, L-FAST still has some shortcomings. First, the point feature extraction is carried out on the edge graph, and the generation of the edge graph increases the time consumption. Although our extraction time was less than that of the FAST algorithm, the total time taken, plus the generation time of the edge graph, was more than that taken by FAST. In addition, L-FAST is obviously not good at pitch angles. The future improvement direction lies in overcoming these problems.

## Figures and Tables

**Table 1:** Comparison of the PSNR and SNR of the two algorithms on four standard digital images.

	Cameraman		Lena		Peppers		Terraux	
	Gaussian	Proposed	Gaussian	Proposed	Gaussian	Proposed	Gaussian	Proposed
SNR	6	7	6	9	6	9	5	10
PSNR	66	73	67	75	69	77	67	95

**Table 2:** The comparison of C/B and C/A values of two algorithms on four standard digital images.

	Cameraman		Lena		Peppers		Terraux	
	C/B	C/A	C/B	C/A	C/B	C/A	C/B	C/A
Canny	0.42	0.22	0.42	0.45	0.45	0.22	0.43	0.15
Proposed	0.25	0.12	0.21	0.18	0.18	0.12	0.18	0.03

**Table 3:** The analysis of absolute trajectory errors.

	RMSE/m	Mean/m	Median/m	Std/m	Min/m	Max/m
FAST	0.80	0.72	0.69	0.33	0.06	1.30
L-FAST	0.76	0.68	0.64	0.33	0.02	1.51

**Table 4:** The RMSE, min and max errors of ATE for sequences from 00 to 10 (unit: m).

		00	01	02	03	04	05	06	07	08	09	10
RMSE	FAST	1.29	9.44	5.61	0.81	0.68	0.8	0.96	0.53	3.47	1.57	1.14
	L-FAST	1.03	10.01	6.23	0.80	0.43	0.76	0.63	1.96	2.98	1.63	4.51
min	FAST	0.10	0.57	0.32	0.06	0.05	0.06	0.07	0.02	0.26	0.09	0.35
	L-FAST	0.09	0.26	0.17	0.02	0.01	0.02	0.02	0.33	0.47	0.04	0.43
max	FAST	3.38	22.59	17.35	1.33	1.16	1.30	1.45	2.56	7.54	4.00	2.82
	L-FAST	2.78	26.19	19.14	1.67	1.58	1.51	1.34	8.04	8.03	4.94	17.51

**Table 5:** The running time of each frame for sequences from 00 to 10 (unit: ms).

	00	01	02	03	04	05	06	07	08	09	10
FAST	31.2	24.3	32.8	31.9	32.5	28.4	25.7	30.1	32.3	31.8	32.1
L-FAST	54.3	32.9	60.7	42.5	51.4	47.1	32.1	68.5	53.9	51.0	85.7

## Data Availability

Not applicable.
